# Indo-Swiss Symposium on Cohorts and Biobanks with special reference to chronic non-communicable diseases

**DOI:** 10.1186/1753-6561-7-S5-A1

**Published:** 2013-08-30

**Authors:** KR Thankappan, Mitchell G Weiss, Probst-Hensch Nicole, K Radhakrishnan

**Affiliations:** 1 Achutha Menon Centre for Health Science Studies, Sree Chitra Tirunal Institute for Medical Sciences and Technology, Trivandrum, India; 2 University of Basel, and former Head, Department of Epidemiology and Public Health, Swiss TPH, Basel, Switzerland; 3 University of Basel, and Department of Epidemiology & Public Health, Swiss Tropical and Public Health Institute, Basel, Switzerland; 4 Sree Chitra Tirunal Institute for Medical Sciences and Technology, Trivandrum, India

## 

An indo-Swiss symposium on cohorts and biobanks with special reference to chronic non-communicable diseases was organized at Sree Chitra Tirunal Institute for Medical Sciences and Technology, Trivandrum, India on 27-28 January 2012. The symposium was sponsored by the Department of Science and Technology (DST), Government of India in the frame work of the Indo-Swiss Joint Research Programme (ISJRP) and organized in association with the Swiss Tropical and Public Health Institute, Basel, Switzerland. The objectives of the symposium were: (1) consider the value and priority of cohorts and bio-banks to explain vulnerability and risk factors, and to guide policy, (2) review Indian and Swiss experience and approaches to establishing and managing various cohorts, (3) clarify cross-cutting implications and prospects for cohort planning in India and Switzerland, and for particular chronic diseases and (4) examine ethical issues to be addressed in design and planning of cohort studies and bio-banking.

